# The sexual health of male sex workers in England: analysis of cross-sectional data from genitourinary medicine clinics

**DOI:** 10.1136/sextrans-2013-051320

**Published:** 2013-11-22

**Authors:** Louise Mc Grath-Lone, Kimberly Marsh, Gwenda Hughes, Helen Ward

**Affiliations:** 1 Department of Infectious Disease Epidemiology, School of Public Health, Imperial College London, London, UK; 2 Department of HIV & STIs, Centre for Infectious Disease Surveillance and Control, Public Health England, London, UK

**Keywords:** Sexual Health, Surveillance, Males, Prostitution

## Abstract

**Objectives:**

Male sex workers (MSW) are thought to be at increased risk of sexually transmitted infections (STI), however, limited comparative data with other groups are available. Disparities among MSWs by migrant status may also exist. Using newly available, cross-sectional surveillance data, the characteristics of MSWs and other male genitourinary medicine (GUM) clinic attendees can be investigated.

**Methods:**

Demographic characteristics, STI prevalence and service usage among MSWs and other male attendees between 1 January and 31 December 2011 were compared using logistic regression.

**Results:**

In 2011, 627 780 men attended GUM clinics; 488 (0.08%) were identified as MSWs. MSWs used a variety of services, however, one in seven had no HIV test at presentation. Adjusting for demographic factors and self-reported sexual orientation, MSWs had increased risk of some STIs and reinfection compared to other male attendees (eg, OR_adj_ of gonorrhoea infection: 2.21, 95% CI 1.61 to 3.01, p<0.001, 14.1% vs 4.8% reinfected in 2011, p=0.005). Service usage did not vary between migrant and UK-born MSWs, but migrant MSWs were twice as likely to be diagnosed with chlamydia.

**Conclusions:**

Some STIs are more prevalent and some reinfections more common among MSWs than other male attendees. A minority of MSWs do not appear to access STI/HIV testing through GUM clinics, and targeted interventions to improve uptake of testing in MSWs should be developed. Service usage and sexual health of MSWs does not appear to vary greatly by migrant status, though the increased risk of chlamydia infection among migrant MSWs should be investigated further.

## Introduction

In the UK, little is known about the characteristics of male sex workers (MSW) and, though they are thought to be at increased risk of sexually transmitted infections (STI),^1^ there are limited comparative data with other groups. Disparities by migrant status in sexual health outcomes and service usage have been described among female sex workers (FSW),^2^ but have not been investigated among MSWs in the UK. Using routinely gathered STI surveillance data that only recently became available we describe the demographic and clinical characteristics of MSWs, their risk of STI acquisition and patterns of service access in comparison to other male genitourinary medicine (GUM) clinic attendees, as well as the variation among MSWs by migrant status.

## Methods

The Genitourinary Medicine Clinic Activity Dataset (GUMCAD) is a patient-level, electronic dataset including diagnoses made and services provided at all GUM clinics in England. Details of the GUMCAD database held by Public Health England (PHE, formerly the Health Protection Agency), guidelines for collecting and reporting GUMCAD data and details of the identifying code for sex workers (SW) have been previously published.^3^ The subset of data for this study was a record of tests, services and diagnoses for all visits to GUM clinics in England by males between 1 January and 31 December 2011. Men recorded as SWs by the application of the SW identifying code to their consultation during at least one visit in 2011 were classified as MSWs for any other visits that year. The demographic characteristics, patterns of attendance, use of services and STI period prevalence for MSWs and other male attendees were compared using Pearson χ^2^ tests. Period prevalence was defined as the proportion of individuals tested for an STI in 2011 who experienced an episode of that STI. The effect of SW status on STI and reinfection was explored using multivariate logistic regression, adjusting for age, ethnicity, sexual orientation, deprivation and clinic location. UK-born and migrant MSWs (defined here as ‘non-UK born’) were similarly compared.

## Results

In 2011, 488/627 780 (0.08%) of male GUM clinic attendees were identified as SWs. MSWs were slightly older than other male attendees (median age; 29 vs 28 years, p=0.05, see web table S1) and were more likely to be migrants (37.1% vs 18.5%, p<0.001) or men who have sex with men (MSM, 57.0% vs 14.8%, p<0.001). The 181 migrant MSWs reported 50 countries of origin, with 38.7% coming from South America (97.1% of these were from Brazil), 24.9% from Europe and 12.2% from Eastern Europe (see web table S2). There was no significant difference in median age between migrant and UK-born MSWs, but migrant MSWs were twice as likely to be recorded as MSM (77.3% vs 35.0%, p<0.001).

MSWs made more visits than other male attendees (mean number of visits in 2011; 4.5 vs 2.3, p<0.001) with migrant MSWs making more visits than UK-born MSWs (5.0 vs 4.4, p=0.03). Visits by MSWs were concentrated in large clinics providing SW-specific services; 86.7% of migrant MSW and 78.3% of UK-born MSW visits were recorded at just five clinics, four of which were in London. A greater proportion of MSWs than other male attendees had a sexual health screen (testing for chlamydia, gonorrhoea and syphilis; 85.5% vs 67.6%, p<0.001) or, where appropriate, a HIV test (86.1% vs 73.1%, p<0.001). MSWs were also more likely to have had post-exposure prophylaxis for HIV following sexual exposure (PEPSE, 4.5% vs 0.4%, p<0.001). There was no significant variation in service usage observed between migrant and UK-born MSWs.

Table 1 compares the period prevalence for STI and HIV among (1) MSWs and other male attendees and (2) UK-born and migrant MSWs. The most prevalent STI diagnosed was chlamydia, with a significantly higher period prevalence observed among MSWs (24.7% vs 9.6%, p<0.001). Adjusting for demographic factors, including self-reported sexual orientation, MSWs were three times more likely to be diagnosed with chlamydia or HIV and twice as likely to be diagnosed with gonorrhoea as other male attendees. Given that MSWs made more visits, on average, than other male attendees, the increased odds of diagnosis among MSWs may have been influenced by the higher number of opportunities they had to be diagnosed. As our model did not adjust for number of visits, we also compared prevalence of STI diagnosis in those tested at first visit and found that MSWs were still almost twice as likely to be diagnosed with chlamydia (3.5% vs 2.0%, p=0.02) and gonorrhoea (0.8% vs 0.4%, p=0.04). Reinfections with chlamydia and gonorrhoea were also more common for MSWs than other male attendees (8.8% vs 3.6% and 14.1% vs 4.8% of those infected respectively, p<0.001). Adjusting for demographic factors, migrant MSWs were twice as likely to be diagnosed with chlamydia as UK-born MSWs (OR_adj_: 2.20, 95% CI 1.08 to 4.49, p=0.03), but there were no statistically significant differences in the prevalence of other STIs or in reinfection (see web table S3).

**Table 1:** Period prevalence of selected STIs (1) among males attending GUM clinics in England in 2011 by sex worker status and (2) among male sex workers attending GUM clinics in England in 2011 by migrant status

(1)	Male sex workers			Other male attendees				Association with being a male sex worker		
	Diagnosed (n)	Tested (n)	Period prevalence (%)	Diagnosed (n)	Tested (n)	Period prevalence (%)	p Value	OR (adjusted*)	95% CI	p Value
Chlamydia‡	113	457	24.7	49 330	514 916	9.6	**<0.001**	2.87	(2.21 to 3.72)	**<0.001**
Gonorrhoea	78	447	17.4	14 155	509 731	2.8	**<0.001**	2.21	(1.61 to 3.01)	**<0.001**
Syphilis	11	421	2.6	2532	450 700	0.6	**<0.001**	1.04	(0.48 to 2.23)	0.93
HIV§	14	383	3.7	2605	440 708	0.6	**<0.001**	3.37	(1.86 to 6.02)	**<0.001**

(2)	UK-born male sex workers			Migrant male sex workers				Association with being a migrant male sex worker		
	Diagnosed (n)	Tested (n)	Period prevalence (%)	Diagnosed (n)	Tested (n)	Period prevalence (%)	p Value	OR (adjusted†)	95% CI	p Value

Chlamydia‡	57	218	26.1	45	172	26.2	0.99	2.20	(1.08 to 4.49)	**0.03**
Gonorrhoea	28	210	13.3	39	170	22.9	**0.02**	0.90	(0.45 to 1.83)	0.78
Syphilis	4	198	2.0	6	157	3.8	0.31	0.75	(0.12 to 4.55)	0.76
HIV§	5	189	2.7	9	130	6.9	0.07	0.96	(0.27 to 3.38)	0.95

Significant differences (p<0.05) are highlighted in bold.

*Adjusting for age, ethnicity, migrant status, sexual orientation, clinic location (inner, outer or outside London) and index of multiple deprivation.

†Adjusting for age, ethnicity, sexual orientation, clinic location (inner, outer or outside London) and index of multiple deprivation.

‡Code suffixes to identify oral or rectal chlamydia infections were introduced to GUMCAD in 2011. However, as the use of these suffixes was not consistent across all clinics in 2011 the data presented here includes all chlamydia infections. In future, it will be possible to provide information on the site of infection.

§New HIV diagnoses in 2011.

GUM, genitourinary medicine; STI, sexually transmitted infection.

## Discussion

Using newly available routine data we have been able to provide a picture of the demographic characteristics and sexual health of MSWs attending GUM clinics in England in 2011. The population was small but diverse, with migrant MSWs originating from a large number of countries. Interestingly, 30% of MSWs were aged ≥35 years, and the median age was 29 years which contrasts with assumptions that MSWs are a predominantly younger group but is consistent with another study of MSWs in Spain.^4^ It may be that older MSWs are more likely to attend GUM clinics and/or disclose they are SWs. The proportion of MSWs who were MSM was low and varied by migrant status. However, it should be noted that MSM status in GUMCAD is based on self-reported sexual orientation, and men recorded as heterosexual are assumed to not be MSM. As MSM status is not always congruent with sexual orientation (ie, MSWs may engage in sex with men, but self-identify as heterosexual^5^), it is possible that the proportion of MSWs who engage in sex with men, and the risks known to be associated with it, have been under-represented. As other statistical analyses of STI risk among MSWs have found mixed effects when adjusting for sexual orientation as a proxy for MSM status^6^ ^7^ the low proportion of MSWs recorded as MSM warrants further exploration.

By comparison with other male GUM clinic attendees, MSWs were significantly more likely to be diagnosed with chlamydia, gonorrhoea and HIV, even when adjusting for key factors, such as age and sexual orientation. However, the prevalence of HIV recorded among MSWs (3.7%) was lower than that of 6.3–14.1% previously reported in MSWs attending a GUM clinic in London.^6^ MSWs were also more likely to experience reinfection with chlamydia and gonorrhoea. Other potential confounders (eg, intravenous drug use (IDU), unprotected anal intercourse with non-paying partners (UPAI)^8^ ^9^) could not be controlled for in our analyses as they are not included in the dataset. Currently, the collection of behavioural data (including UPAI and IDU) through GUMCAD is being piloted by PHE, and this may enable the impact of these factors to be explored in the future.

MSWs were significantly more likely than other attendees to have a sexual health screen or HIV test; however, these were not recorded for all MSWs. The one in eight MSWs who had no sexual health screen, or the one in seven who had no HIV test recorded, may reflect major missed opportunities. Though uptake is high in comparison to other male attendees, further work to identify the barriers to STI and HIV testing, and improve uptake among MSWs attending clinics, should be a priority. High levels of reinfection with chlamydia and gonorrhoea experienced by MSWs should also be explored.

In this study, differences in sexual health and service usage among MSWs in the UK by migrant status were investigated for the first time. While chlamydia infection was more common among migrant MSWs, there was no difference in other STIs or reinfections, in the proportion accessing sexual health screens or HIV testing through GUM clinics, or in the proportion that made repeated use of these services. While missing data on country of birth means these results must be interpreted cautiously, it appears that, by contrast with FSWs, UK-born and migrant MSWs have similar sexual health issues and patterns of service use. The increased risk of chlamydia infection among migrants should, however, be explored further. By comparison with the 2003 population of MSWs attending a London GUM clinic described by Sethi *et al*^6^, a greater proportion of our MSWs were UK-born or from South America. In the future, as data continues to be gathered routinely through GUMCAD, trends in the composition of the changing population of men engaged in sex work in England will be available in a timely manner, and may be useful for planning tailored, language-appropriate MSW services.

As a routine STI surveillance system, GUMCAD has limitations; for example, it does not currently report on risk factors known to be associated with STI acquisition in MSWs, and patient records cannot be matched and de-duplicated across clinics. Furthermore, it provides a convenience sample of MSWs attending GUM clinics who may not be representative of the wider MSW population. It is also likely that GUMCAD underestimates the number of MSWs attending GUM clinics, due in part to a lack of disclosure. UK studies have reported that as few as one-third of SWs disclose their status to healthcare workers.^10^ The way in which SW status is ascertained and recorded by individual GUM clinics may also be a contributing factor. A survey of GUM clinics we conducted showed variation in who is asked about sex work, how they are asked and how this information is recorded. This means that the identifying SW code may not always be applied during the subsequent coding of the consultation. Nonetheless, our study provides a national picture of the comparative sexual health and service use of MSWs attending GUM clinics, allowing their specific sexual health needs to be explored. It also uses routinely gathered mandatory surveillance data making it cheaper and easier than special SW studies requiring recruitment of MSWs.

## Supplementary Material

Web supplement
