# Supplementary material for: The sexual health of male sex workers in England: analysis of cross-sectional data from genitourinary medicine clinics
Source: Sex Transm Infect. 2013 Nov 22;90(1):38–40. doi: 10.1136/sextrans-2013-051320 (PMC3913221; doi:10.1136/sextrans-2013-051320)
Supplement: Web supplement [file sextrans-2013-051320-s1.pdf]

Web table 1: Socio-demographic characteristics of and use of services by males attending GUM clinics in England in 2011 by sex worker status

		Male sex workers (N=488)		Other male attendees (N=627,292)		
Socio-demographic characteristics		n	%	n	%	p-value
Age*	<19	16	*3.3*	55,168	*8.8*	
	20-24	105	*21.5*	157,211	*25.1*	
	25-29	130	*26.6*	133,322	*21.3*	
	30-34	90	*18.4*	89,110	*14.2*	
	35-44	94	*19.3*	107,164	*17.1*	
	45+	52	*10.7*	82,216	*13.6*	**<0.001**
	Median	29 years		28 years		**0.05**
Ethnicity†	White	307	*62.9*	455,740	*72.7*	
	Mixed	30	*6.1*	19,184	*3.1*	
	Asian or Asian British	12	*2.5*	30,752	*4.9*	
	Black or Black British	45	*9.2*	61,239	*9.8*	
	Other	40	*8.2*	14,781	*2.4*	**<0.001**
Sexual orientation‡	Heterosexual	197	*40.4*	475,206	*75.8*	
	MSM	278	*57.0*	92,746	*14.8*	**<0.001**
Migrant status¥	U.K. born	237	*48.6*	470,140	*74.9*	
	Non-U.K. born	181	*37.1*	116,056	*18.5*	**<0.001**
Services used						
	Sexual health screen	417	*85.5*	423,853	*67.6*	**<0.001**
	HIV test¶	383	*86.1*	440,708	*73.1*	**<0.001**
	Vaccination (hepatitis B)§	65	*13.8*	23,739	*3.8*	**<0.001**
	PEPSE	22	*4.5*	2,750	*0.4*	**<0.001**

*Age was unknown for 1 male sex worker and 101 other male attendees. †Ethnicity was unknown for 54 male sex workers and 45,596 other male attendees. ‡Sexual orientation was unknown for 13 male sex workers and 59,340 other male attendees. ¥Migrant status was unknown for 70 male sex workers and 41,096 other male attendees. ¶Of those appropriate to test for HIV i.e. not recorded as known HIV positive or attending for HIV related care at first visit in 2011 (n=445 for male sex workers and n=602,840 for other male attendees) §Of those appropriate to receive hepatitis vaccination i.e. not diagnosed with hepatitis B or recorded as known hepatitis B immune in 2011 (n=470 for male sex workers and n=620,738 for other male attendees). PEPSE= Post-exposure prophylaxis for HIV following sexual exposure. MSM = Men who have sex with men

Web table 2: Socio-demographic characteristics of and use of services by male sex workers attending GUM clinics in England in 2011 by migrant status

		UK born MSW (N=237)		Migrant MSW (N=181)		
Socio-demographic characteristics		n	%	n	%	p-value
Age*	<19	10	*4.2*	3	*1.7*	
	20-24	51	*21.5*	38	*21.0*	
	25-29	59	*24.9*	55	*30.4*	
	30-34	41	*17.3*	39	*21.5*	
	35-44	42	*17.7*	35	*19.3*	
	45+	34	*14.3*	10	*5.5*	**0.04**
	Median	29 years		29 years		0.70
Ethnicity[†]	White	174	*73.4*	102	*56.4*	
	Mixed	15	*6.3*	15	*8.3*	
	Asian or Asian British	3	*1.3*	8	*4.4*	
	Black or Black British	29	*12.2*	12	*6.6*	
	Other	3	*1.3*	28	*15.5*	**<0.001**
Sexual orientation[‡]	Heterosexual	147	*62.0*	36	*19.9*	
	MSM	83	*35.0*	140	*77.3*	**<0.001**
Region of birth (for migrants)	U.K.			n/a	n/a	
	Europe[¥]			45	*24.9*	
	Eastern Europe			22	*12.2*	
	Africa			13	*7.2*	
	Asia			23	*12.7*	
	Australia			3	*1.7*	
	North America			5	*2.8*	
	South America			70	*38.7*	n/a
Services used						
	Sexual health screen	196	*82.7*	155	*85.6*	0.42
	HIV test[¶]	189	*84.7*	130	*84.4*	0.93
	Vaccination (hepatitis B)[§]	23	*10.2*	23	*13.1*	0.37
	PEPSE	7	*3.0*	11	*6.1*	0.12

*Age was unknown for 1 migrant male sex worker (MSW). [†]Ethnicity was unknown for 13 UK born and 16 migrant MSWs. [‡]Sexual orientation was unknown for 7 UK born and 5 migrant MSWs. [¥]Europe excludes UK and Eastern Europe. [¶]Of those appropriate to test for HIV i.e. not recorded as known HIV positive or attending for HIV related care at first visit in 2011 (n=223 for UK born and n=154 for migrant MSWs) [§]Of those appropriate to receive hepatitis vaccination i.e. not diagnosed with hepatitis B or recorded as known hepatitis B immune in 2011 (n=226 for UK born and n=176 for migrant MSWs). PEPSE= Post-exposure prophylaxis for HIV following sexual exposure. MSM = Men who have sex with men

Web Table 3: Number of males diagnosed with STIs of interest through GUM clinics in 2011 stratified by sex worker and migrant status and sexual orientation

	Other male attendees	All MSWs	UK born MSWs	Migrant MSWs
**Chlamydia**				
Heterosexual	38,802	63	46	12
MSM	7,135	49	10	33
Not specified	3,393	1	1	0
Total	49,330	113	57	45
**Gonorrhoea**				
Heterosexual	5,922	13	10	3
MSM	7,063	64	18	35
Not specified	1,170	1	0	1
Total	14,155	78	28	39
**Syphilis**				
Heterosexual	455	0	0	0
MSM	1,922	10	3	6
Not specified	155	1	1	0
Total	2,532	11	4	6
**HIV**				
Heterosexual	859	0	0	0
MSM	1,515	14	5	9
Not specified	231	0	0	0
Total	2,605	14	5	9
